# Ginsenoside Rg5 modulates the TLR4 and BCL-2 pathways by inhibiting NOX1, thereby alleviating inflammation, apoptosis and pyroptosis in hyperuricemia nephropathy

**DOI:** 10.1016/j.jgr.2025.03.009

**Published:** 2025-03-25

**Authors:** Yu-Xin Zhang, Hui Wan, Guan-Yue Shan, Jun-Ya Cheng, Zhi-Cheng Gao, Yi-Ying Liu, Wen-Na Shi, Zi-Jun Sun, Hai-Jun Li

**Affiliations:** a Department of Transplantation Immunology, Institute of Translational Medicine, The First Hospital of Jilin University, Changchun, Jilin Province, China; b Department of Bioengineering, Pharmacy School of Jilin University, Changchun, Jilin Province, China; c Department of Biopharmacy, Pharmacy School of Jilin University, Changchun, Jilin Province, China

**Keywords:** Hyperuricemia nephropathy, Ginsenoside Rg5, NOX1, Inflammation, Pyroptosis, Apoptosis

## Abstract

**Background:**

Hyperuricemia nephropathy (HN) is a form of renal injury caused by hyperuricemia, which can progress to chronic kidney disease (CKD) and end-stage renal disease (ESRD). Ginsenoside Rg5, a major bioactive compound isolated from Panax ginseng, is recognized for its notable effects, including anti-inflammatory, antioxidant, and anticancer activities.

**Method:**

The toxic doses of MSU crystals and Rg5-induced HK-2 cell damage were assessed using the CCK-8 assay and quantifying oxidative stress markers (MDA, GSH, SOD). Intracellular stress was evaluated with JC-1 and DCFH-DA probes. Bioinformatics analysis identified NOX1, TLR4, and Bcl-2 as potential targets. The protein expression associated with stress, inflammation, pyroptosis, and apoptosis in HK-2 cells was evaluated through a combination of Western blotting, ELISA, flow cytometry, immunofluorescence, and overexpression methods. An HN mice model was established through administration of YE and adenine, and the effects of Rg5 were evaluated. The in vivo mechanisms were further verified.

**Results:**

Rg5 reduced serum uric acid, BUN, ADH, and creatinine levels in MSU crystals-stimulated HK-2 cells and hyperuricemic mice, alleviating renal damage. Rg5 inhibited NOX1 and suppressed the TLR4 pathway, reducing oxidative stress, inflammation, pyroptosis, and apoptosis. NOX1 overexpression reversed the effects of Rg5, while TLR4 overexpression had no effect. Rg5's efficacy was similar to NOX1 inhibitor ML171.

**Conclusion:**

These results indicate that Rg5 can modulate the TLR4 and BCL-2 pathways by inhibiting NOX1, thereby alleviating oxidative stress, inflammation, pyroptosis, and apoptosis in HN, highlighting its potential as a therapeutic approach for controlling HN.

## Highlights

• This study investigated the role of NOX1 in HN for the first time.
• Rg5 inhibits high NOX1 expression, easing inflammation, apoptosis, and pyroptosis.
• This study demonstrates the therapeutic potential of Rg5 in HN.

## Graphical abstract

**Figure:**
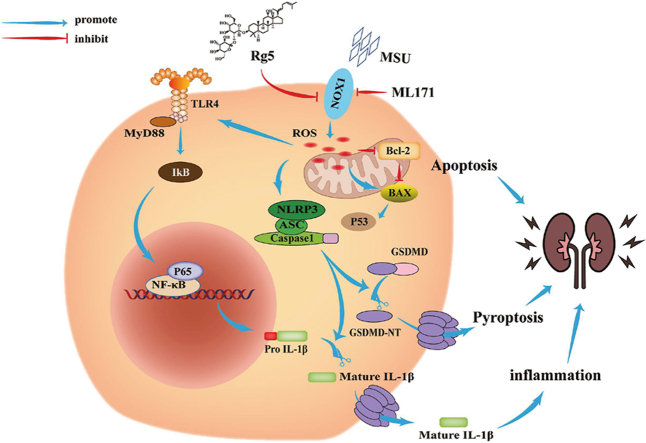

## 1 Introduction

Hyperuricemia is a prevalent metabolic condition that is characterized by increased levels of uric acid in the blood [1]. Uric acid (UA), which is the final byproduct of purine metabolism, is typically regulated by the kidneys and various other mechanisms to ensure that proper levels are maintained in the bloodstream [2]. The excessive production or inadequate excretion of UA leads to its accumulation in the body. Symptoms may occur due to genetic metabolic defects, impaired renal function, drug side effects, or other factors [3]. The incidence of hyperuricemia has been increasing. Chronic hyperuricemia can lead to gout, cardiovascular, and renal damage [4]. When UA levels exceed its solubility, UA crystals form and are deposited in joints, soft tissues, and kidneys. The kidneys are the primary organs responsible for the excretion and reabsorption of UA. When UA levels increase and accumulate in the kidneys, monosodium urate (MSU) crystals may form, leading to both crystal-dependent and crystal-independent kidney damage. Chronic UA deposition and inflammation can lead to the progression of chronic kidney disease, which is marked by a gradual deterioration of kidney function and may ultimately result in kidney failure. Investigating the pathogenesis of hyperuricemia nephropathy (HN) and identifying effective treatment strategies are crucial. Oxidative stress and inflammation are closely associated with the progression of HN [5]. Oxidative stress occurs due to an imbalance between cellular antioxidants and the antioxidant system [6]. Excess reactive oxygen species (ROS) levels can trigger inflammatory responses, mitochondrial apoptosis, and chronic kidney disease (CKD) progression [7]. Reducing ROS production may alleviate oxidative stress and improve kidney function [8,9].

The development of HN involves multiple proteins and signaling pathways. NADPH oxidase 1 (NOX1) plays a crucial role as a source of oxidative stress. NOX1 increases the levels of intracellular ROS by oxidizing NADPH to produce superoxide, which subsequently leads to downstream damage [10]. NOX1 is associated with to various renal disorders, including hyperlipidemic renal injury, diabetic nephropathy, and ischemia-reperfusion injury [[11], [12], [13]]. Although many studies have linked NOX1 to various renal diseases, its role in HN has rarely been discussed. Toll-like receptor 4 (TLR4), which is a member of the TLR family that is expressed in mammalian cells, performs crucial functions in identifying pathogen-related molecular patterns and in activating the innate immune system [14]. TLR4, which is a crucial receptor in mammalian cells, is very important for alleviating renal injury through the inhibition of the TLR4/MyD88/NF-κB signaling pathway [[15], [16], [17]]. NOD-like receptor protein 3 (NLRP3) inflammasome, which is a key component of innate immunity, is implicated in various inflammatory diseases [18]. Under the action of the NLRP3 inflammasome, Gasdermin D (GSDMD) is cleaved into GSDMD-N. GSDMD-N is an effector protein that can trigger cell death and cytokines release [19,20]. Numerous studies have also shown that NOX1 can induce cell apoptosis [[21], [22], [23]]. These observations further suggest that the TLR4 signaling pathway and apoptosis can interact with NOX1 and are associated with the inflammatory response in HN.

Current treatments for hyperuricemia focus on lowering blood UA levels. However, drugs such as febuxostat, colchicine, and benzbromaron, which are used to lower UA levels may cause side effects, including cardiovascular risks, skin reactions, and liver damage [24]. Increasing evidence suggests the efficacy of herbs and natural products in treating gout and HN with fewer side effects. In ancient times, ginseng (*Panax ginseng* C.A. Mey) was used to treat HN [25]. Ginsenoside, which is the main active ingredient of ginseng, has been extensively studied because of its wide range of pharmacological effects. These effects include anti-inflammatory, antitumor, antiobesity, and antiviral effects as well as beneficial effects on the cardiovascular system [[26], [27], [28], [29]]. Ginsenosides are classified into common ginsenosides and rare ginsenosides (RGSs), with RGS, such as Rg5, exhibit increased anticancer activity and better absorption [30]. Rg5 has a diverse range of pharmacological activities, such as anti-inflammatory, anticancer, and antidermatitis activities, and it has the ability to enhance memory [[31], [32], [33]]. It also suppresses inflammatory factors in models of cisplatin-induced acute kidney injury caused by cisplatin and diabetic nephropathy. Moreover, Rg5 alleviates cisplatin-induced nephrotoxicity by reducing inflammation, oxidative stress, and apoptosis [[34], [35], [36]]. Recent studies have indicated that RGS enriched with Rg5 can inhibit xanthine oxidase activity, restore kidney function, and regulate gut microbiota balance to improve hyperuricemia [41]. The therapeutic potential of Rg5 in HN remains largely unexplored, thus comprehensive investigation of its specific mechanisms is warranted. In this study, we first validated the therapeutic effects of Rg5 on MSU crystals-stimulated HK-2 cells in both in vitro and in vivo experiments. We then explored the specific changes in HK-2 cells under HN conditions using pathway-related inhibitors, activators, and overexpression strategies, to further evaluate the potential mechanisms through which Rg5 alleviates HN.

## 2 Materials and methods

### 2.1 Reagents and antibodies

Rg5 (≧98 %, 186763-78-0) Beijing Beinachuanglian Biotechnology Research Institute (Beijing, China). Monosodium urate crystals (MSU, HY-B2130A), ML171 (HY-12805) and Resatorvid (TAK-242) were purchased from Med Chem Express (New Jersey, USA). Antibodies for NOX1 (FNab05803), URAT1 (FNab09285), GLUT9 (FNab10270) and GSDMD (FNab03670) were purchased from Fine Test (Wuhan, China). Antibodies for MyD88 (4283), IĸB (4812), NF-ĸB p65 (5114), P53 (2527), Bcl-2 (4223) and Bax (2772) were derived from Cell Signaling Technology (MA, USA). Antibodies including ABCG2 (222011), OCT-2 (R382304), OAT1 (252608), TLR4 (505258), NLRP3 (381207), p65(380172), ASC (340097), Caspase-1 (342947) and IL-1β (516288) were supplied by ZEN-BIOSCIENCE (Chengdu, China). Antibody for GADPH (TA802519) was purchased from ORIGENE (Wuxi, China). PE anti-human CD284 (TLR4, 312806) was purchased from BioLegend (CA, USA). Peroxidase-conjugated goat anti-rabbit immunoglobulin G (IgG) (H + L) (AP31L117) and anti-mouse IgG (H + L) (AP31L119) were purchased from Life-iLab (Chengdu, China). CCK-8 test kit (C0037), ROS test kit (S0033S), JC-1 staining test kit (C2006), yeast extract (YE, ST968), PI staining test kit (ST511) and Hoechst 33258 staining test kit (C1011) were purchased from Beyotime Biotechnology (Shanghai, China). Allopurinol (Allop, A800424) and Adenine (A800684) were purchased from Mackin (Shanghai, China). The assays for measuring Aspartate aminotransferases (AST, C010-2-1), Alanine aminotransferases (ALT, C009-2-1), Lactate dehydrogenase assay kit (LDH, A020-2-2),Glutathione (GSH, A006-2-1), Superoxide dismutase (SOD, A001-3-2), Malondialdehyde (MDA, A003-4-1), Uric acid Test Kit (UA, C012-2-1), Blood Urea nitrogen Kit (BUN, C013-2-1), Creatinine Assay kit (CRE, C011-2-1), Urea Assay Kit (UN, C013-2-1), Xanthine Oxidase assay kit (XOD, A002-1-1) and Adenosine deaminase assay kit (ADA, A048-2-1) were conducted using kits obtained from Nanjing Jiancheng Bioengineering Institute (Nanjing, China). Annexin V-FITC/PI Apoptosis kit (E-CK-A211) was purchased from Elabscience (Wuhan, China).

### 2.2 Cell culture and treatment

Human kidney 2 (HK-2, CL-0109, Pricella, Wuhan, China) cells were cultured in Dulbecco's Modified Eagle Medium (DMEM) with 10 % fetal bovine serum (FBS) and 1 % penicillin-streptomycin and maintained at 37 °C in 5 % CO_2_. Upon achieving complete adherence to the culture vessel, the cells were proceeded to the subsequent experimental steps. For the evaluation of Rg5 cytotoxicity, cells were exposed to various concentrations of Rg5 (0, 0.2, 1, 5, 10, 20, 40, 60, 80 μM) for 24 h. Similarly, MSU crystals-induced cytotoxicity was assessed by treating cells with MSU crystals (0, 0.05, 0.1, 0.2, 0.4, 0.8, 1 mg/ml) for 24 h. To investigate the protective effect of Rg5 against MSU crystals-induced hyperuric acid renal injury, HK-2 cells were pre-incubated with various concentrations of Rg5 (0.2, 1, 5 μM) for 6 h prior to exposure to MSU crystals (0.1 mg/ml) for 24 h. To assess the effect of NOX1 inhibition on cellular responses and Rg5 treatment, we chose to add ML171 (2 μM) 1 h before Rg5 treatment. To assess the impact of TLR4 inhibition on cell responses and Rg5 treatment, we chose to administer Resatorvid (5 μM) 1 h prior to Rg5 treatment.

### 2.3 Animals

Male C57BL/6 mice (6–8 weeks old) were purchased from Jilin Qianhe Model Biotechnology Co., Ltd. (Changchun, China), quarantined, and acclimated for 1 week. All animal experiments were approved by the Animal Care and Use Committee of the First Hospital of Jilin University (Approval No.20231022). The mice were housed at 20–23 °C and 40–80 % humidity with free access to food and water.

### 2.4 Establishment and treatment of HN model

The dosing regimen for the animal experiments in this study was based on relevant literature [37,38]. The protocol for inducing the HN model involved randomly dividing wild-type (WT) mice into five groups (n = 5): Control (normal saline, i.g.), model (YE 15 g/kg + ad 100 mg/kg, i.g.), Rg5 low-dose (Rg5 10 mg/kg, i.p.), Rg5 high-dose (Rg5 20 mg/kg, i.p.), and allop (allopurinol 10 mg/kg, i.g.). YE, adenine, Rg5, and allop were dissolved in 0.9 % normal saline. The Rg5 low-dose and high-dose groups were pre-treated for three days and injected once a day. The mice in the Rg5 and allop groups were respectively given Rg5 (10, 20 mg/kg) and allopurinol (10 mg/kg) by gavage 6 h after the Rg5 and allopurinol administrations, the mice were administered Rg5 (10 and 20 mg/kg) and allop (10 mg/kg) using a 15 g/kg YE + 100 mg/kg adenine to establish a model of hyperuricaemia-induced renal injury. The experimental procedure was repeated once a day for 1 week. Body weight was recorded daily during the experiment. On the 8th day, the mice were put into metabolic cages for 12 h to collect urine with fasting but free access to water. On day 9, blood was collected from the eyeballs of all experimental mice, and kidney tissues were collected by decapitation treatment. Serum was separated by centrifugation at 6000 g for 10 min at 4 °C. The weights of kidneys were recorded. The kidney weight was recorded and the renal coefficient (the ratio of kidney weight to body weight) was calculated. Kidney tissue was fixed in 4 % paraformaldehyde or used to prepare kidney tissue homogenate supernatant.

### 2.5 Western blot (WB)

HK-2 cells were lysed in RIPA buffer with PMSF at a 100:1 ratio. Then, centrifugation was conducted at 12,000 rpm for 10 min to obtain the supernatant. The protein concentration was measured by a BCA assay (Beyotime, P0012). Protein samples were boiled for denaturation, separated via 10 %–15 % SDS-PAGE, and transferred onto a PVDF membrane. After being blocked with 5 % milk in PBST for 1 h, the membrane was incubated with primary antibodies overnight at 4 °C. Subsequently, after washing with PBST, the secondary antibodies were added for 1 h at room temperature. Finally, grayscale value analysis was carried out.

### 2.6 Statistical analysis

Data were analyzed with GraphPad Prism 9.5, and results are expressed as mean ± standard deviation (SD). One-way analysis of variance (ANOVA) was used for multiple comparisons, and each experiment was repeated thrice to ensure statistical robustness. Statistical significance was indicated as follows: (#P < 0.05, ##P < 0.01, ###P < 0.001 vs. Control group; ∗P < 0.05, ∗∗P < 0.01, ∗∗∗P < 0.001 vs. MSU or model group).

## 3 Result

### 3.1 Rg5 reduced UA transport and reverse MSU crystals-induced HK-2 cell damage

**Fig. 1:**
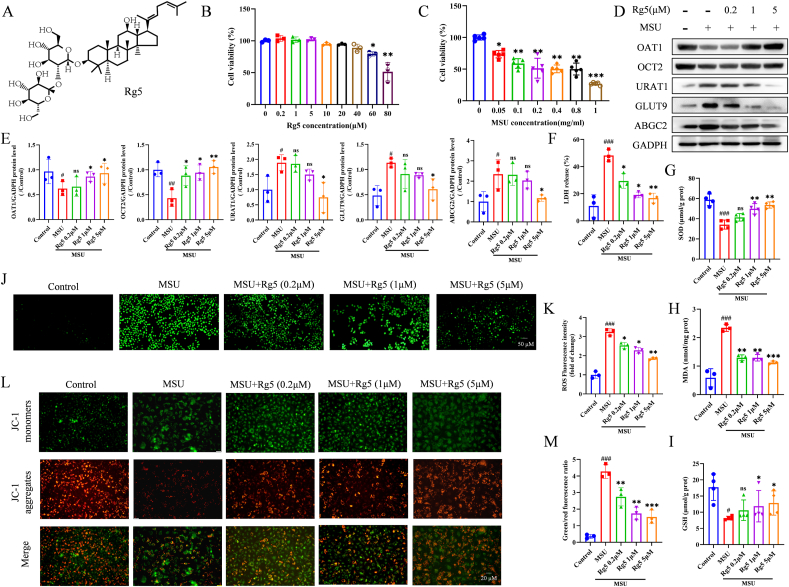
**Rg5 had the ability to lower uric acid and reverse the HK-2 cell damage caused by MSU**. (A) Chemical structure of Rg5. (B) Effect of Rg5 on cell viability of HK-2 cells, n = 3. (C) Effect of MSU on cell viability of HK-2 cells s, n = 3. (D–E) Representative western blotting images of OAT1, OCT2, URAT1, GLUT9 and ABGC2 in HK-2 cells with or without MSU stimulation, after treatment with different concentrations of Rg5. (E) Changes in the relative protein expression levels of OAT1, OCT2, URAT1, GLUT9 and ABGC2, n = 3. (F) The cell lysate was collected to analyze lactate dehydrogenase (LDH) secretion, n = 3. Effects of Rg5 on the levels of SOD (G), MDA (H) and GSH (I) in MSU-activated HK-2 cells, n = 3. (J–K) ROS production was measured by DCFH-DA staining in HK-2 cells (scale bar, 50 μm), statisitics of the relativity of ROS fluorescence, n = 3. (L–M) The HK-2 cell were stained with JC-1 to measure MMP, and the MMP were calculated by the ratio of red/green (scale bar, 20 μm), n = 3. ^#^P < 0.05, ^##^P < 0.01, ^###^P < 0.001 vs. Control group; ∗P < 0.05, ∗∗P < 0.01, ∗∗∗P < 0.001 vs. MSU group.

Hyperuricemia may lead to imbalanced excretion and re-absorbance of UA and then an excessive accumulation of MSU crystals in the kidney, which consequently resulted in kidney injury. Fig. 1A depicts the molecular structure of Rg5. The lowest cytotoxic dose of Rg5 and the optimal MSU dose for simulating HN were determined using the CCK-8 assay. Concentrations of 0.2, 1 and 5 μM of Rg5 and 0.1 μM of MSU were selected for subsequent experiments (Fig. 1B–C).

Induction using 0.1 μM MSU crystals led to a remarkable impairment of the UA transport cycle. We assessed the changes in urate transporter proteins associated with UA excretion. The expression of URAT1, GLUT9, and ABCG2 was increased, while the expression of OAT1 and OCT2 was decreased. Rg5 treatment caused a dose-dependent decrease in URAT1, GLUT9, and ABCG2 expression and reversed the decline of OAT1 and OCT2 expression (Fig. 1D–E).

Rg5 dose-dependently reduced LDH secretion compared to the MSU group (Fig. 1F). The effect of Rg5 on oxidative stress was evaluated through measuring the intracellular levels of SOD (Fig. 1G), MDA (Fig. 1H), and GSH (Fig. 1I). Under MSU crystals treatment, MDA levels rose while SOD and GSH activities decreased. However, Rg5 significantly reversed the induced oxidative stress. Fluorescence analysis indicated that Rg5 also reduced ROS levels (Fig. 1J–K) and mitigated MSU crystals-induced mitochondrial damage (Fig. 1L–M). These results suggest that Rg5 effectively mitigates UA transport and reverses MSU crystals-induced damage in HK-2 cells.

### 3.2 Rg5 attenuated MSU crystals-induced HN by regulating NOX1 expression

**Fig. 2:**
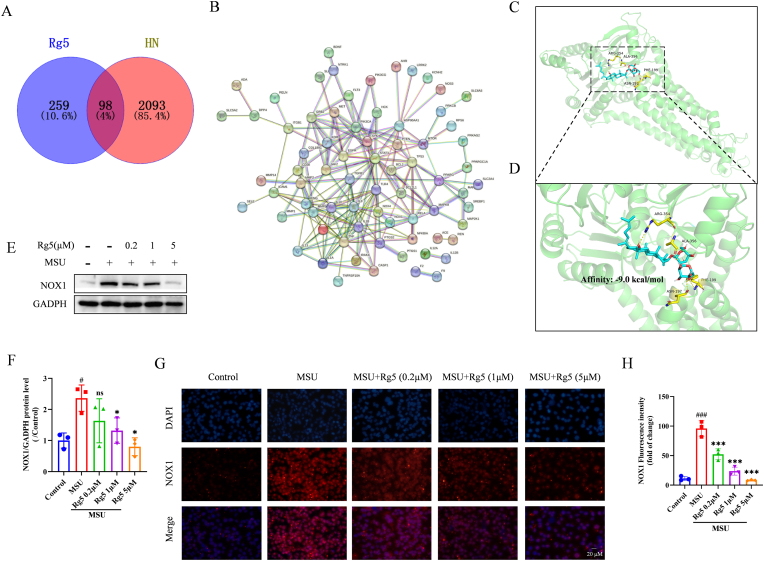
**Rg5 inhibited the expression of NOX1 stimulated by MSU**. (A) Venn diagram of screened Rg5 acting on HN targets. (B)The STRING database generates a network of potential targets for Rg5 interaction with HN. (C–D) Molecular docking results of Rg5 and NOX1. (E) Representative western blotting images of NOX1 with or without MSU stimulation, after treatment with different concentrations of Rg5. (F) Changes in the relative protein expression levels of NOX1, n = 3. (G–H) The expression levels of NOX1 were assessed by immunofluorescence (scale bar, 20 μm), n = 3. ^#^P < 0.05, ^###^P < 0.001 vs. Control group; ∗P < 0.05, ∗∗∗P < 0.001 vs. MSU group.

Network pharmacology analysis was performed to explore the molecular mechanism of Rg5 in the HN model. A total of 357 Rg5-targeting compounds were identified using Swiss Target Prediction and Super-PRED, while 2191 HN-related targets were retrieved from GeneCards, HERB, DisGeNET, and OMIM. The intersection of Rg5 targets and HN-related targets identified 98 common targets (Fig. 2A). An interaction network for these targets was created using the STRING database (Fig. 2B). Notably, the TLR4, BCL-2, and NOX1 proteins were identified as potentially significant.

We identified that Rg5's potential mechanism in treating HN might involve NOX1. Molecular docking revealed direct hydrogen-bonding interactions between Rg5 and NOX1 at ARG354, ALA356, PHE199, and ASN197 (Fig. 2C–D). These findings suggest NOX1 as a target in HN treatment, with Rg5 potentially reducing MSU crystals-induced HN by modulating NOX1 expression. WB confirmed that NOX1 expression was elevated in the MSU group compared to Control, and was dose-dependently reduced following Rg5 treatment (Fig. 2E–F). IF showed consistent results (Fig. 2G–H). Additionally, we further confirmed the direct interaction between Rg5 and NOX1 through fluorescence co-localization (Fig. S1-A-D). In conclusion, data suggest NOX1 is involved in HN pathogenesis and Rg5 alleviates MSU crystals-induced HN by inhibiting it.

### 3.3 Rg5 inhibited MSU crystals-induced HK-2 cells inflammatory and pyroptosis

**Fig. 3:**
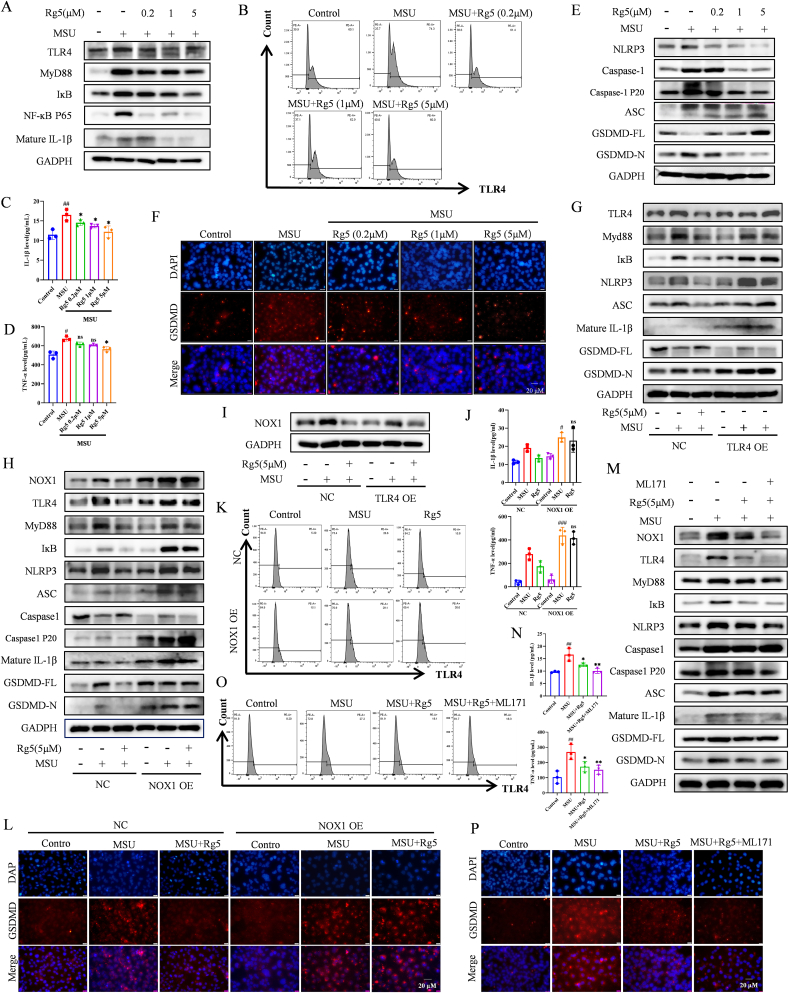
**Rg5 inhibited the activation of the TLR4/NLRP3 signaling pathway and pyroptosis induced by MSU crystal stimulation in a NOX1-dependent manner**. HK-2 cells treated with different concentrations of Rg5 under conditions with or without MSU stimulation (A–F). HK-2 cells were transfected with control (pcDNA3.1) or NOX1 OE (pcDNA3.1(+)-human NOX1) for 48 h and then stimulated with MSU for 24 h (H, J-L). HK-2 cells were transfected with control (pcDNA3.1) or TLR4 OE (pcDNA3.1(+)-human TLR4) for 48 h and then stimulated with MSU for 24 h (G, I). HK-2 was treated with or without ML171 (2 μM) for 1 h, followed by Rg5 and MSU for 24 h (M − P). (A) Representative Western blot images of TLR4, MyD88, IκB, NF-κB P65, pro IL-1β and mature IL-1β proteins in HK-2 cells. (B) Flow cytometry detection of TLR4 levels. (C–D) The release of mature IL-1β and TNF-α in culture supernatant was detected by ELISA, n = 3. (E) Representative western blotting images of NLRP3, Caspase-1, Caspase-1 P20, ASC, GSDMD-FL, and GSDMD-N in HK-2 cells. (F) The expression levels of GSDMD were assessed by immunofluorescence (scale bar, 20 μm). (G, I) Representative Western blot images of TLR4, MyD88, IκB, NF-κB P65, pro IL-1β, mature IL-1β, and NOX1 in HK-2 cells. (H, M) Representative Western blot images of NOX1, TLR4, MyD88, IκB, NLRP3, Caspase-1, Caspase-1 P20, ASC, pro IL-1β, mature IL-1β, GSDMD-FL, and GSDMD-N proteins. (J, N) The release of mature IL-1β and TNF-α in culture supernatant was detected by ELISA, n = 3. (K, O) Flow cytometry detection of TLR4 levels. (L, P) The expression levels of GSDMD were assessed by immunofluorescence (scale bar, 20 μm). #P < 0.05, ##P < 0.01, ###P < 0.001 vs. Control group; ∗P < 0.05, ∗∗P < 0.01, ∗∗∗P < 0.001 vs. MSU group.

TLR4 signaling facilitates the activation of NLRP3 inflammasome, thereby leading to pyroptosis in the kidney. Compared to the Control, MSU crystals increased TLR4, MyD88, IκB, and NF-κB p65, mature IL-1β with dose-dependent decreases following Rg5 treatment (Fig. 3A, Fig. S2A). Flow cytometry showed increased TLR4 in MSU crystals-treated cells, which recovered after Rg5 treatment (Fig. 3B). Given the central role of TNF-α in inflammation, we measured both TNF-α and IL-1β using ELISA to assess the anti-inflammatory effects. Rg5 decreased IL-1β and TNF-α expression during inflammatory vesicle activation (Fig. 3C–D). MSU crystals stimulation upregulated pyroptosis-related proteins, which Rg5 pretreatment reversed (Fig. 3E, Fig. S2B). GSDMD acts as the effector in pyroptosis, and is essential in host defense and danger signaling. IF confirmed that GSDMD increased in MSU crystals-stimulated HK-2 cells and was downregulated by Rg5 (Fig. 3F, Fig. S2C). In the TLR4 OE group, MSU crystals significantly increased inflammation and pyroptosis-related proteins, while Rg5's therapeutic effect was impaired (Fig. 3G, Fig. S2D). These findings indicate that Rg5 alleviates the inflammation and pyroptosis induced by MSU crystals in HK-2 cells.

### 3.4 Rg5 inhibited activation of the TLR4/NLRP3 signaling pathway and pyroptosis in a NOX1-dependent manner

To further explore NOX1's role in inflammation and pyroptosis during Rg5-mediated MSU crystals-induced HN alleviation, we used NOX1 overexpression plasmid (Fig. S3A-B) and ML171 (NOX1 inhibitor, Fig. S3C-D) in the following experiments. NOX1 pcDNA3.1(+) plasmid was transfected into HK-2 cells, revealing that NOX1 OE blocked Rg5's inhibitory effects on TLR4 activation, pyroptosis, and IL-1β release (Fig. 3H, Fig. S4A). Rg5 and ML171 similarly inhibited MSU crystals-induced TLR4/NLRP3 activation (Fig. 3M, Fig. S4D). While TLR4 OE did not block Rg5's inhibition of NOX1 (Fig. 3I, Fig. S4B), NOX1 OE inhibited Rg5's effect on TLR4 (Fig. 3H, Fig. S4A). These findings suggest that TLR4/NLRP3 is a downstream pathway of NOX1 in Rg5 treatment. ELISA of IL-1β and TNF-α, flow cytometry for TLR4 and GSDMD expression, and IF staining yielded consistent results (Fig. 3J–L, N-P, Fig. S4C and E). In addition, we included a TLR4 inhibitor, Resatorvid, for a more comprehensive validation. As shown in Fig. S5 of the revised manuscript, Resatorvid (5 μM) treatment effectively suppressed TLR4 and its downstream signaling pathways, with minimal effect on NOX1 expression. Overall, these results indicate that Rg5 inhibits TLR4/NLRP3 activation and pyroptosis in a NOX1-dependent manner.

### 3.5 Rg5 inhibited MSU crystals-induced HK-2 cells apoptosis in a NOX1-dependent manner

**Fig. 4:**
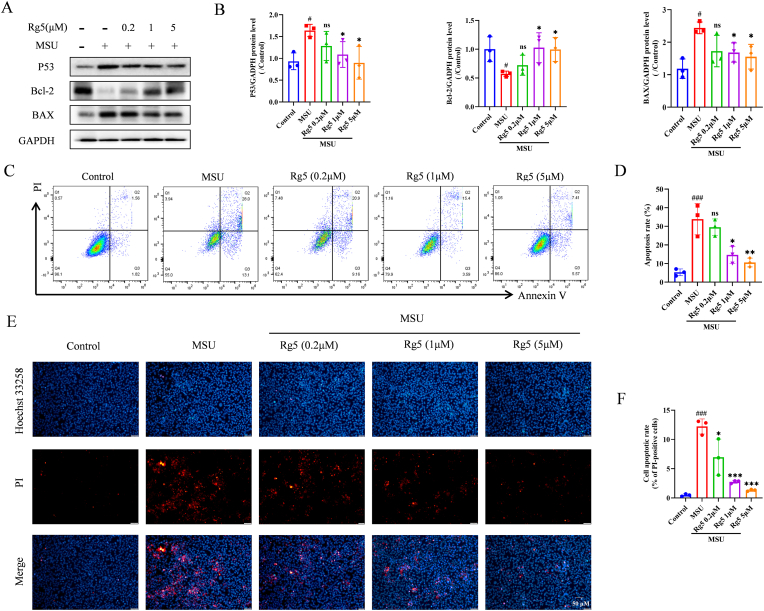
**Rg5 inhibited MSU induced HK-2 cell apoptosis**. (A) Representative western blotting images of P53, Bcl-2 and BAX with or without MSU stimulation, after treatment with different concentrations of Rg5. (B) Changes in the relative protein expression levels of P53, Bcl-2 and BAX, n = 3. (C–D) Rg5 inhibited HK-2 cells apoptosis induced by MSU, as analyzed by flow cytometry with PI and Annexin V antibodies. (E–F) Cells were stained with PI (2 μg/ml) and Hoechst 33342 (5 μg/ml) for 10 min, and the percentage of PI-positive (red) cells relative to total cells (Hoechst 33342, blue) was analyzed (Scale bar, 50 μm), n = 3. #P < 0.05, ###P < 0.001 vs. Control group; ∗P < 0.05, ∗∗P < 0.01, ∗∗∗P < 0.001 vs. MSU group.

Since oxidative stress has a role in mediating apoptosis, we explored if Rg5 could regulate the apoptosis - related changes in MSU crystals-stimulated HK-2 cells. In MSU crystals-treated HK-2 cells, the ratio of monomer/aggregate was increased, indicating a significant increase in oxidative stress-induced MMP (mitochondrial membrane potential) reductio. However, Rg5 treatment alleviated mitochondrial oxidative stress and stabilized MMP. This suggests that Rg5 scavenges ROS and alleviates oxidative stress (Fig. 1J–M). As expected, treating HK-2 cells with MSU crystals for 24 h raised the levels of P53 and BAX and reduced Bcl-2. But pretreatment with Rg5 reversed these changes in a dose-dependent manner (Fig. 4A–B). Flow cytometry was used to confirm the anti-apoptotic effects of Rg5 (Fig. 4C–D). Hoechst 33342/PI staining also demonstrated that exposure to MSU crystals notably enhanced PI uptake, and this effect was reversed by pretreatment with Rg5 (Fig. 4E–F). Therefore, Rg5 effectively modulated the mitochondrial apoptotic pathway and reduced apoptosis in the MSU crystals-stimulated HK-2 cell model.

**Fig. 5:**
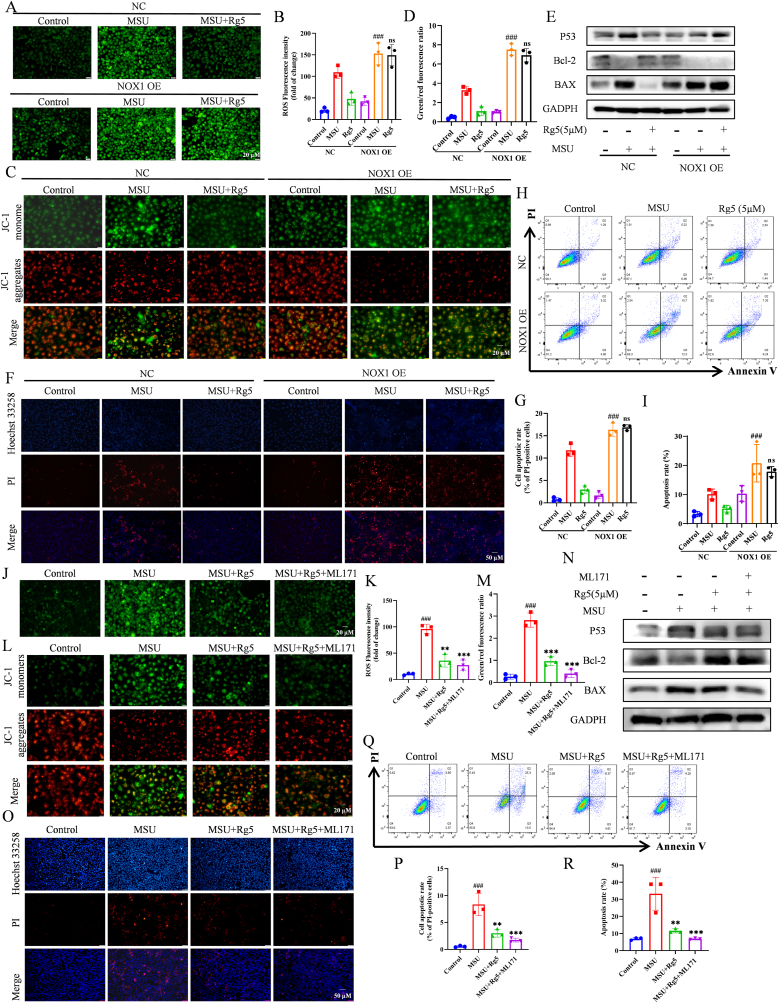
**Rg5 exerts an ameliorative effect on HK - 2 cell apoptosis induced by MSU stimulation in a NOX1-dependent manner**. HK-2 was transfected with sh-NC (pcDNA3.1) or sh-NOX1 (pcDNA3.1(+)-human NOX1) for 48 h and then stimulated with MSU for 24 h, where Rg5 was selected at a high concentration (5 μm) (A–I). HK-2 was treated with or without ML171 (2 μM) for 1 h, followed by Rg5 and MSU for 24 h, where Rg5 was selected at a high concentration (5 μm) (J–R). (A-B, J-K) ROS production was measured by DCFH-DA staining in HK-2 cells (scale bar, 20 μm), statisitics of the relativity of ROS fluorescence, n = 3. (C-D, L-M) The HK-2 cell were stained with JC-1 to measure MMP, and MMP were calculated by the ratio of red/green (scale bar, 20 μm), n = 3. (E, N) Representative Western blotting images of P53, Bcl-2 and BAX. (F-G, O-P) Cells were stained with PI and Hoechst 33342, and the percentage of PI-positive (red) cells relative to total cells (Hoechst 33342, blue) was analyzed (Scale bar, 50 μm), n = 3. (H-I, Q-R) Analyzed by flow cytometry with Annexin V and PI antibodies, n = 3. #P < 0.05, ###P < 0.001 vs. Control group; ∗∗P < 0.01, ∗∗∗P < 0.001 vs. MSU group.

To explore the mechanism of NOX1 regulation of apoptosis in Rg5-mediated alleviation of MSU crystals-induced HN, we used two complementary approaches. NOX1 pcDNA3.1(+) plasmid was transfected into HK-2 cells to assess its effects on oxidative stress, ROS levels, and MMP alterations. Compared to Control, the NOX1 OE-MSU and NOX1 OE-Rg5 groups exhibited elevated ROS and reduced MMP levels (Fig. 5A–D). This suggests that NOX1 overexpression exacerbated oxidative stress and reversed Rg5's therapeutic effect. As shown in Fig. 5E and Fig. S6A, in the NOX1 OE-MSU group, Bcl-2 expression decreased, while P53 and BAX levels increased, and no further improvement after Rg5 treatment. Hoechst33258/PI staining and Annexin V FITC/PI flow cytometry results were consistent with WB analysis (Fig. 5F–I). We also used ML171 to inhibit NOX1 in the Rg5 group. The results indicated similar inhibition in the Rg5 and ML171 groups, with enhanced apoptosis suppression in the ML171+Rg5 group (Fig. 5J–R, Fig. S6B). In summary, Rg5 inhibited MSU crystals-induced mitochondrial oxidative stress and apoptosis in a NOX1-dependent manner.

### 3.6 Rg5 ameliorated hyperuricemia kidney injury in HN mice

**Fig. 6:**
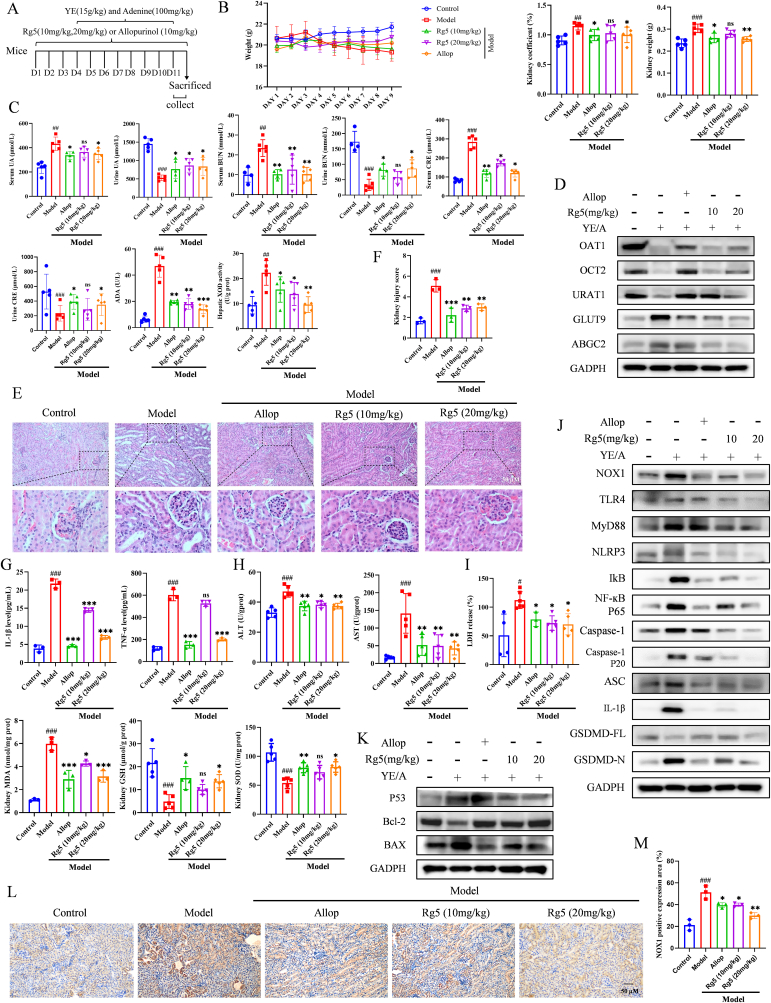
**Rg5 attenuates uric acid kidney injury in a NOX1-dependent manner in hyperuricemic nephropathic mice**. (A) The experimental design in vivo. (B) Effect of Rg5 and allopurinol on body weight, renal coefficient and kidney weight, n = 5. (C) Serum UA, CRE, BUN, XOD, ADA and urine UA, CRE, BUN levels in mice kidney, n = 3. (D) Representative Western blotting images of OAT1, OCT2, URAT1, GLUT9 and ABGC2 in mice kidney. (E) H&E staining from mice kidney in the indicated groups (scale bar = 50 μm). (F) Kidney injury scores, n = 3. (G) Levels of mature IL-1β and TNF-α were detected by ELISA kits, n = 3. Measurement of serum ALT, AST(H), LDH, GSH, MDA and SOD(I), n = 3. (J–K) Representative Western blotting images of NXO1, TLR4, MyD88, NLRP3, IĸB, NF-ĸB P65, Caspase-1, Caspase-1 P20, ASC, Pro IL-1β, Mature IL-1β, GSDMD-FL, GSDMD-N, P53, Bcl-2 and BAX in mice kidney. (L–M) Immunohistochemistry staining of NOX1 from mice in the indicated groups and quantitative analyses of NOX1 protein expression (scale bar = 50 μm), n = 3. #P < 0.05, ##P < 0.01, ###P < 0.001 vs. Control group; ∗P < 0.05, ∗∗P < 0.01, ∗∗∗P < 0.001 vs. Model group.

The effects of Rg5 on hyperuricemia kidney injury in vivo were assessed in a HN model induced by 8 days of YE and adenine gavage following a 3-day pre-treatment with Rg5. The in vivo experimental design was outlined in Fig. 6A. After 8-day treatment, the model group had significant increases in kidney weight, renal coefficient and serum UA, BUN, CRE, ADA, XOD levels, and decreases in urine UA, BUN, CRE levels. However, Rg5 (10 and 20 mg/kg) and allop significantly reversed these changes (Fig. 6B–C). Furthermore, Rg5 (10 and 20 mg/kg) and allop reversed protein expression changes, decreasing URAT1, GLUT9, and ABCG2, and restoring OAT1 and OCT2 levels (Fig. 6D, Fig. S7A).

As depicted in Fig. 6E, the results of HE staining indicated that the Control group had a normal renal structure. In contrast, the model group presented severe renal injury, which encompassed inflammatory cell aggregation, glomerular damage, tubular dilation, vacuolar degeneration, and necrosis. Both Rg5 and allop protected against renal pathological changes induced by hyperuricemia. Renal injury scores correlated with pathomorphological changes, with Rg5 and allop significantly improving these scores (Fig. 6F). These results suggest that Rg5 alleviated UA-induced renal injury and attenuated pathomorphological changes in HN mice.

### 3.7 Rg5 attenuated symptoms in a NOX1-dependent manner in HN mice

We further investigated whether Rg5 could alleviate mouse HN by inhibiting NOX1. Detection in kidney homogenates showed the model group had elevated TNF - α, IL - 1β, ALT, AST, LDH, MDA levels and decreased GSH, SOD activities. Treatment with Rg5 (10, 20 mg/kg) or allop reversed the changes in these markers, while Rg5 (20 mg/kg) and allop reversed the YE/adenine-induced decline in GSH and SOD activities (Fig. 6G–I). WB analysis confirmed that Rg5 (10, 20 mg/kg) or allop (10 mg/kg) inhibited the activation of the NOX1/TLR4 and NOX1/Bcl-2 pathway, consistent with in vitro findings (Fig. 6J–K, Fig. S7B-C). Immunohistochemistry revealed that the model group upregulated NOX1 expression, which was reduced by Rg5 or allop (Fig. 6L–M). Overall, results suggest Rg5 modulates the TLR4 and BCL-2 pathways by inhibiting NOX1, thereby alleviating inflammation, apoptosis, and pyroptosis in HN in vivo.

## 4 Discussion

The kidney serves as the principal organ for excreting UA, accounting for 70 percent of the systemic excretion [39]. HN, a CKD subtype, features disrupted purine metabolism and elevated serum UA levels. [40]. Due to the lack of targeted therapies, HN remains a global health challenge, highlighting the need for new treatments. Ginsenosides, a component of Ginseng, have shown nephroprotective effects [41]. But the therapeutic role of Rg5 in HN and its underlying mechanisms remain unclear. This study explored the effects of Rg5 on HN and its underlying mechanisms, providing insights for therapeutic strategies. Rg5 protected against HN by reducing oxidative stress, inflammation, pyroptosis, and apoptosis.

In this study, the HN mice model was induced by 8 days of oral YE/adenine administration, followed by biochemical and histopathological analyses of renal function and pathology. Zhang et al. [42] found ginsenosides reduced UA, urea nitrogen in hyperuricemic mice. HN mice had higher UA, CRE, BUN, XOD, ADA levels than Controls. Renal tubular enlargement and vacuolar lesions were evident in HN mice, confirming successful model establishment. Renal dysfunction and injury were significantly ameliorated by Rg5 treatment in a dose-dependent manner. Rg5 dose-dependently reduced serum UA, CRE, BUN, XOD, and ADA levels, suggesting that Rg5 improved renal damage in mice. To explore the underlying mechanisms of Rg5's protective effect, we established a 0.1 mg/ml MSU crystals-induced HK-2 cell injury model for in vitro studies. This study demonstrated that Rg5 improves HN by inhibiting the NOX1/TLR4 and NOX1/BCL-2 pathways, and its interaction with NOX1 was confirmed through molecular docking and fluorescence co-localization.

HN involves oxidative stress and inflammatory injury as key factors [43]. Oxidative stress activates immune responses and increases cytokine production to activate downstream transcription factors [44]. NOXs are transmembrane proteins which generate O_2_ radicals and ROS by transferring electrons [45]. Elevated inflammatory cytokines stimulate free radicals, enhancing oxidative stress and perpetuating renal injury [5]. In this study, Rg5 significantly reduced NOX1, ROS, IL-1β, TNF-α, MDA, LDH, and restored SOD and GSH levels in HN mice kidneys. These results suggest that Rg5 prevents HN-related oxidative stress in kidney tissues, consistent with in-vitro findings. Previous studies have shown that NOX1 expression and activity are elevated in kidney tissues of inflammatory disease models like hypertension, ischemia-reperfusion injury, hyperuricaemia, diabetes, and hypercholesterolemia [12,13]. Therefore, Rg5 plays a key role in preventing hyperuricaemia-induced kidney damage via oxidative stress.

NOXs play a key role in regulating inflammatory responses following TLR activation [46], and can activate the NLRP3 inflammasome to promote the maturation of pro-IL-1β [19,47]. A large number of studies have also shown that NOX1 can act as an upstream protein in the activation of the NLRP3 inflammasome [48]. The TLR4 signaling pathway and NLRP3 inflammasome are critical mediators of inflammation [49,50]. TLR4 activation triggers NF-κB signaling, leading to increased transcription of NLRP3 and IL-1β. Subsequently, the NLRP3 inflammasome cleaves pro-IL-1β to generate mature IL-1β and GSDMD-N. These pathways are expressed in renal intrinsic, endothelial, and mesangial cells, playing a pivotal role in inflammation and pyroptosis [19,51]. In the context of HN, TLR4 and NLRP3 signaling are involved in renal inflammation [20,52]. Reducing TLR4 expression can mitigate renal dysfunction and pathology in HN [49]. Previous studies have shown that Rg5 modulates TLR4 signaling and NLRP3 inflammasome activation, reducing IL-1β and GSDMD-N levels, and thereby alleviating inflammation and pyroptosis [35,53]. In both in vivo and in vitro experiments, Rg5 was found to downregulate TLR4 and NLRP3 expression, while inhibiting GSDMD-N activation. Furthermore, the inhibitory effect of Rg5 on inflammation and pyroptosis was reversed by NOX1 OE treatment and enhanced by ML171. It is noteworthy that TLR4 OE treatment did not significantly affect the inhibitory effect of Rg5 on NOX1, and the inhibition of NOX1 by Rg5 was not altered by treatment with the TLR4 inhibitor Resatorvid. These findings suggest that Rg5 inhibits TLR4/NLRP3 signaling and cleavage by suppressing NOX1 and reducing ROS production.

Hyperuricemia-induced apoptosis, along with oxidative stress, inflammation, and pyroptosis, contributes significantly to renal injury [54]. Mitochondria-dependent apoptosis, a major mechanism, is caused by excessive ROS disrupting mitochondrial structure and function in proximal tubular epithelial cells. [55,56]. The Bcl-2 family, including Bcl-2 and Bax, regulates mitochondrial apoptosis by forming heterodimers that prevent Bax activation [57]. In this study, increased ROS production resulted in reduced MMP and apoptosis in both in vitro and in vivo models, characterized by elevated P53 and Bax levels, and decreased Bcl-2, consistent with previous findings [58]. These results suggest that the Bcl-2-associated apoptosis pathway contributes to HN, and Rg5 may protect against HN by inhibiting apoptosis through this pathway. The involvement of NOX1 inhibition in this effect was further investigated using NOX1 OE and ML171. Rg5 alleviated HN-induced apoptosis, while NOX1 OE reversed its protective effect. Conversely, ML171 enhanced Rg5's protective action, indicating that Rg5's anti-apoptotic effects are linked to NOX1 signaling activation.

## 5 Conclusion

Our study reveals that Rg5 can alleviate inflammatory injury, oxidative stress, pyroptosis and apoptosis in HN. It is probable that these effects are mediated via the TLR4 and BCL-2 pathways by inhibiting NOX1. These results indicated that Rg5 might be a promising candidate for HN.

## CRediT authorship contribution statement

Yu-xin Zhang and Hai-jun Li developed the study concept and were responsible for the study design. The experiments were performed by Yu-xin Zhang, Zhi-cheng Gao, Hui Wan, Guan-yue Shan, Jun-ya Cheng, Yi-ying Liu, Wen-na Shi and Zi-jun Sun. Yu-xin Zhang wrote the original manuscript and analyzed the data. Hai-jun Li revised the manuscript. All authors contributed to the article and approved the submitted version.

## Declaration of competing interest

The authors declare that they have no known competing financial interests or personal relationships that could have appeared to influence the work reported in this paper.
