# Supplementary material for: Ginsenoside Rg5 modulates the TLR4 and BCL-2 pathways by inhibiting NOX1, thereby alleviating inflammation, apoptosis and pyroptosis in hyperuricemia nephropathy
Source: J Ginseng Res. 2025 Mar 25;49(4):426–37. doi: 10.1016/j.jgr.2025.03.009 (PMC12223446; doi:10.1016/j.jgr.2025.03.009)

**Ginsenoside Rg5 modulates the TLR4 and BCL-2 pathways by inhibiting NOX1, thereby alleviating inflammation, apoptosis and pyroptosis in hyperuricemia nephropathy**

Yu-Xin Zhang^a^, Hui Wan^a^, Guan-Yue Shan^a,b^, Jun-Ya Cheng^a,b^, Zhi-Cheng Gao^a^, Yi-Ying Liu^a,c^, Wen-Na Shi^a,c^, Zi-Jun Sun^a,b^, Hai-Jun Li^a*^

**Supplementary file**

**Materials and methods**

*Over expression of NOX1 or TLR4 in HK-2*

HK-2 cells were plated in 6-well plates (8×105 cells/well) and then transfected with 50 ng plasmid or 500ng plasmid using Lipofectamine 2000 kit (Thermo Scientific, USA) according to the manufacturer’s guidelines for 6h. The pcDNA3.1(+)-human NOX1 (PEGFP-N-5: TGGGAGGTCTATATAAGCAGAG) and the pcDNA3.1(+)-human TLR4 was ordered from IBSBIO (China). The culture medium was replaced by a medium with or without Rg5 (5 μM) for 48 h. After treatment, the cells were washed twice before being used in subsequent procedures.

*Cell viability detection*

HK-2 cells were plated in 96-well plates and allowed to adhere. Following this, the cells were grouped and treated with drugs according to the experimental design. Cell viability was measured using the CCK-8 assay.

*ROS, MMP and apoptosis detection*

Intracellular ROS production was measured using a ROS detection kit with the DCFH-DA probe. Mitochondrial membrane potential (MMP) was assessed using JC-1 staining, and apoptosis was detected with Hoechst33258/PI (PI 2 μg/ ml, Hoechst 33342 5 μg/ml) staining. Fluorescent microscopy was employed to capture images of ROS MMP and apoptosis.

*Measurement of biochemical indicators*

MDA, GSH and SOD levels in kidney tissue and cell pellet were assessed using specific detection kits. LDH, UA, BUN, CRE, XOD and ADA levels in serum and cell pellet were assessed using specific detection kits. Protein concentration in tissues and cells was quantified using the BCA method, with adjustments made prior to experimentation. Detection reagent was added to the supernatant following the kit instructions, followed by an appropriate incubation period, and measurement of the optical density (OD) value.

*Flow Cytometry*

Flow cytometry was applied to detect the levels of TLR4 and cell apoptosis in HK-2. HK-2 was digested with trypsin to prepare the cell suspension. The cells were centrifuged at 1500 rpm for 4 min, then the supernatant was discarded followed by washing with flow buffers twice. PE anti-human CD284 (TLR4, cat #: 312806, BioLegend, CA, USA) or Annexin V-FITC/PI (Elabscience, Wuhan, China) was added and incubated at room temperature for 20 min. The cells were washed twice with flow buffers and the supernatant was discarded. The precipitates were suspended in 300 μL flow buffers and detected by flow cytometry.

*Immunofluorescence staining (IF)*

1. HK-2 cells were seeded into a 12-well plate and allowed to adhere overnight. Subsequently, the cells were fixed and permeabilized. Cells were incubated with a blocking solution containing 5% bovine serum albumin (BSA) for 30 min and washed three times with phosphate-buffered saline with Tween 20 (PBST). The cells were then subjected to overnight incubation at 4°C with pecific primary antibodies (1:150 for NOX1 and 1:100 for GSDMD). The cells were washed thrice with PBST and exposed to fluoresce labeled secondary antibodies after incubation. 4',6-diamidino-2-phenylindole (DAPI) was applied for nuclear counterstaining after 1 h incubation at room temperature in darkness. The expression of NOX1 and GSDMD was visualized using a fluorescence microscope (Olympus).
2. Small Molecule Immunofluorescence Co-localization: First, the FITC-labeled Rg5 compound (FITC-BSA-Rg5), synthesized by Sangon Biotech, which is conjugated with BSA and the FITC fluorophore, will be used. HK-2 cells will be seeded overnight in a 20mm Glass Bottom Cell Culture Dish (Nest, No: 801001) to achieve 70% confluence. The growth medium will be removed, and the cells will be incubated with 5 µM FITC-BSA-Rg5 at 37°C for 6 hours. As described in the supplementary methods for IF, cells will be fixed with 4% paraformaldehyde for 15 minutes, followed by treatment with 0.1% Triton X-100/PBS and washing with PBS three times for 5 minutes each. The cells will then be incubated overnight at 4°C with the primary antibody (NOX1, diluted 1:150). After washing three times with PBST, the cells will be incubated with a fluorophore-conjugated secondary antibody. Nuclear staining will be performed using DAPI at room temperature under light-protected conditions. Finally, imaging will be performed using an Olympus fluorescence microscope.
3. NOX1 Block Immunofluorescence: Similar to the small molecule immunofluorescence co-localization experiment, HK-2 cells will be seeded overnight in a 12-well plate to achieve 70% confluence. The cells will then be co-incubated with 5 µM FITC-BSA-Rg5. After incubation, the growth medium will be removed, and the cells will be treated as described in the IF method. After fixation, cell permeabilization, and PBS washes, the cells will be incubated overnight at 4°C with Anti-NOX1 antibody (abcam, ab55831, diluted to 1 µg/mL). Following three PBST washes, the cells will be incubated with a fluorophore-conjugated secondary antibody. DAPI will be used for nuclear staining under light-protected conditions, and imaging will be performed on an Olympus fluorescence microscope.

*Enzyme-linked immunosorbent assay (ELISA)*

IL-1β and TNF-α (Thermo Scientific, USA) expression in cells were detected with the corresponding Elisa Kits, according to the manufacturers’ instructions.

*Bioinformatics analysis*

Preliminary prediction of potential targets, pathways and mechanisms associated with anti-HN activity in Rg5 using network pharmacology. The therapeutic targets of Rg5 were identified by searching the keywords “Ginsenoside Rg5” in the following databases: SwissTargetPrediction (http://www.swisstargetprediction.ch/); and Super-PRED (https://predic tion.charite.de/index.php). To obtain the Gene Official Symbol format, the targets were imported into the Retrieve/ID mapping function of Uniprot (https ://www.uniprot.org/id-mapping). We screened the GeneCards (https://www.genecards.org/), HERB (http://herb.ac.cn), DisGeNET (https://disgenet.com/) and OMIM (https://www.omim.org/) databases to identify HN-related genes using the keyword "Hyperuricemic nephropathy". To identify potential targets associated with both Rg5 and HN, we utilized an online tool (https://bioinfogp.cnb.csic.es/tools/venny/index.html) for creating venn diagrams.

The STRING database (http://string-db.org/) was utilized to build a protein-protein interaction (PPI) network, setting a minimum interaction score of 0.9 (highest confidence) as a requirement.

*Molecular Docking*

A molecular docking study was conducted to explore the feasible interaction model of Rg5 against NOX1 using the AutoDock Vina software. The 3D structure of NOX1 (SMTL ID: 8wej.1) was downloaded from the SWISS-MODEL databases (https://swissmodel.expasy.org/), which was set as receptor. The 3D structure of Rg5 (PubChem CID: 11550001) was retrieved from the PubChem database (https://pubchem.ncbi.nlm.nih.gov/), and thereafter exploited as ligands. Top ranked poses with the lowest energy score were considered privileged binding model, which were selected for further study. And the docking results were visualized by Pymol software (version 1.6, http://www.pymol.org).

*Histopathological examination*

The kidney tissue specimens were initially fixed in 4% paraformaldehyde solution and subsequently embedded in paraffin to prepare tissue sections. Histological evaluation of liver morphology was conducted using hematoxylin and eosin (HE) staining technique. Microscopic examination of the stained sections was performed under a light microscope.

*Immunohistochemistry*

For immunohistochemical analysis, paraffin sections were dewaxed and rehydrated. After antigen repair, endogenous peroxidase was inactivated with 3% H2O2/methanol for 15 min. Sections were then incubated with 5% BSA and sealed for 1 h, followed by overnight incubation with an anti-NOX1 antibody (1:200, cat #: DF8684, Affinity Biosciences) at 4°C. After incubation with the secondary antibody, sections were stained with DAB and hematoxylin and then sealed for imaging. Images were acquired using a microscope (AVIBENT, Japan).

*Abbreviations*

Hyperuricemia nephropathy (HN); chronic kidney disease (CKD); Monosodium urate crystals (MSU); [Malondialdehyde](https://www.bio-review.com/tag/malondialdehyde/) (MDA); Glutathione (GSH); Superoxide dismutase (SOD); reactive oxygen species (ROS); Uric acid (UA); NOX1 (NADPH oxidase 1); Nicotinamide Adenine Dinucleotide Phosphate (NADPH); Toll-like receptor 4 (TLR4); NOD-like receptor protein 3 (NLRP3); Gasdermin D (GSDMD); rare ginsenosides (RGS); Lactate dehydrogenase (LDH); Myeloid differentiation primary response gene 88 (MyD88); Inhibitor of kappa B (IκB); Nuclear Factor kappa B p65 subunit (NF-κB p65); Interleukin 1 beta (IL-1β); Tumor Necrosis Factor alpha (TNF-α); Mitochondrial membrane potential (MMP); Tumor Protein 53 (P53); Bcl-2-associated X protein (BAX); B-cell lymphoma 2 (Bcl-2); Propidium Iodide (PI); Blood Urea nitrogen (BUN); Creatinine Assay (CRE); Adenosine deaminase (ADA); Xanthine Oxidase (XOD); Urea Assay (UN); Uric acid (UA); Urate transporter 1 (URAT1); Glucose transporter 9 (GLUT9); ATP-binding cassette sub-family G member 2 (ABGC2); Organic anion transporter 1 (OAT1); Organic cation transporter 2 (OCT2); Alanine aminotransferase (ALT); Aspartate aminotransferase (AST); Allopurinol (Allop); Adenine (Ad).

**Fig. S1. Rg5 may directly interact with NOX1 in HK-2 cells.** (A) IF detection was performed on MSU-stimulated HK-2 cells with or without FITC-BSA-Rg5 treatment. Green represents the FITC-BSA-Rg5 compound that interacts with NOX1 protein, red indicates the intracellular expression of NOX1, and blue shows the cell nuclei stained with DAPI. The IF images reveal that FITC-BSA-Rg5 and NOX1 co-localize in specific regions of the treated cells. No such overlap is observed when FITC alone is incubated with the cells (scale bar, 20μm). (B) IF detection was performed on FITC-BSA-Rg5-treated HK-2 cells with or without Anti-NOX1 treatment. Green represents the FITC-BSA-Rg5 compound that interacts with NOX1 protein, red indicates the intracellular expression of NOX1, and blue shows the cell nuclei stained with DAPI. The IF images demonstrate that, compared to the control group, the fluorescence intensity of FITC-BSA-Rg5 and NOX1 inside the cells is significantly reduced after NOX1 blockade.


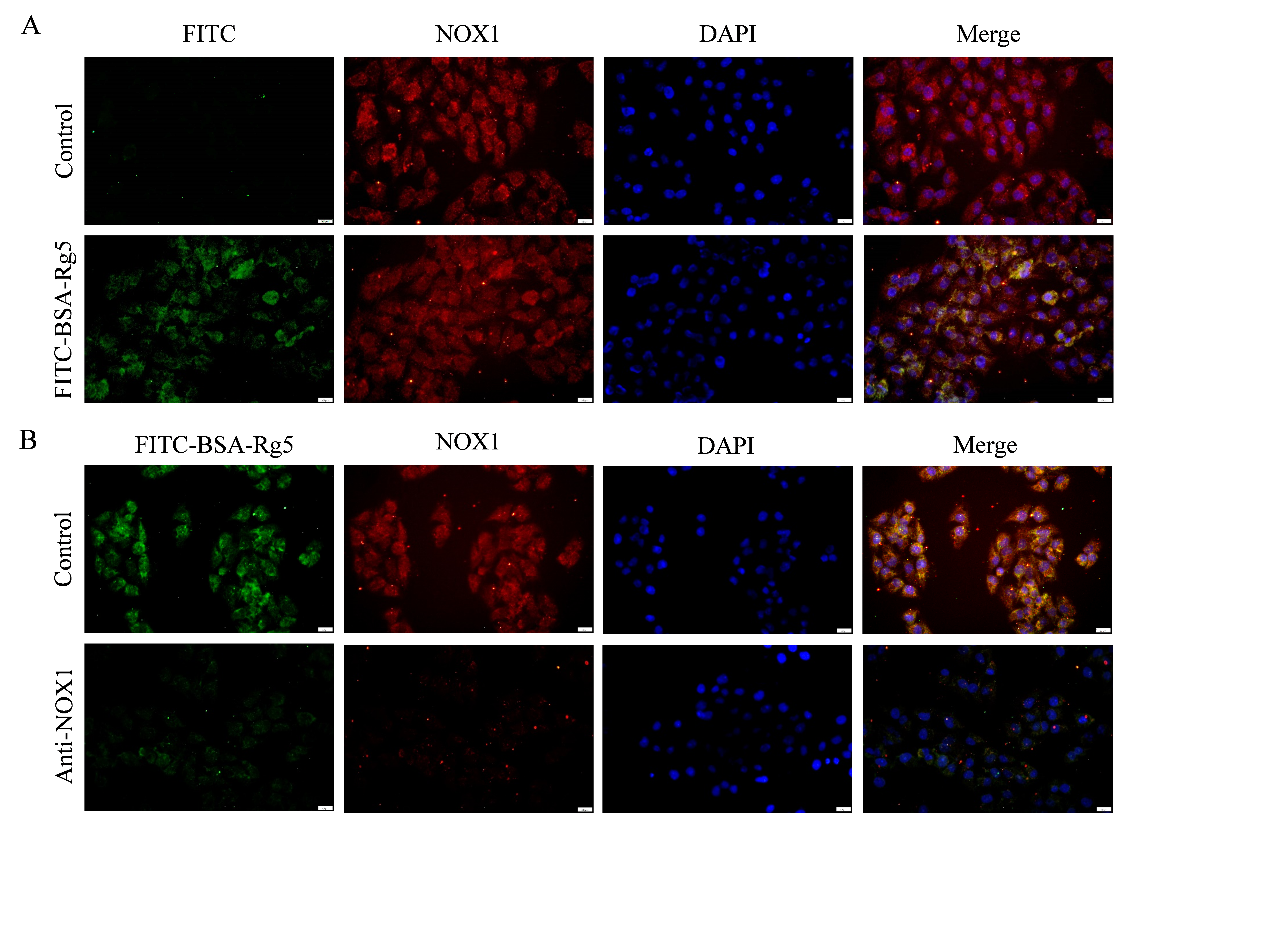


**Fig. S2. Some statistics in Fig. 3.** (A) Changes in the relative protein expression levels of TLR4, MyD88, IĸB, mature IL-1β, n=3. (B) Changes in the relative protein expression levels of NLRP3, Caspase-1, Caspase-1 P20, ASC, GSDMD-FL and GSDMD-N, n=3. (C) IF analysis of GSDMD expression, n=3. (D) Changes in the relative protein expression levels of TLR4, MyD88, IĸB, NLRP3, Caspase-1, Caspase-1 P20, ASC, mature IL-1β, GSDMD-FL and GSDMD-N, n=3. #P<0.05, ##P<0.01, ###P<0.001 vs. Control group; *P<0.05, **P<0.01, ***P<0.001 vs. MSU group.


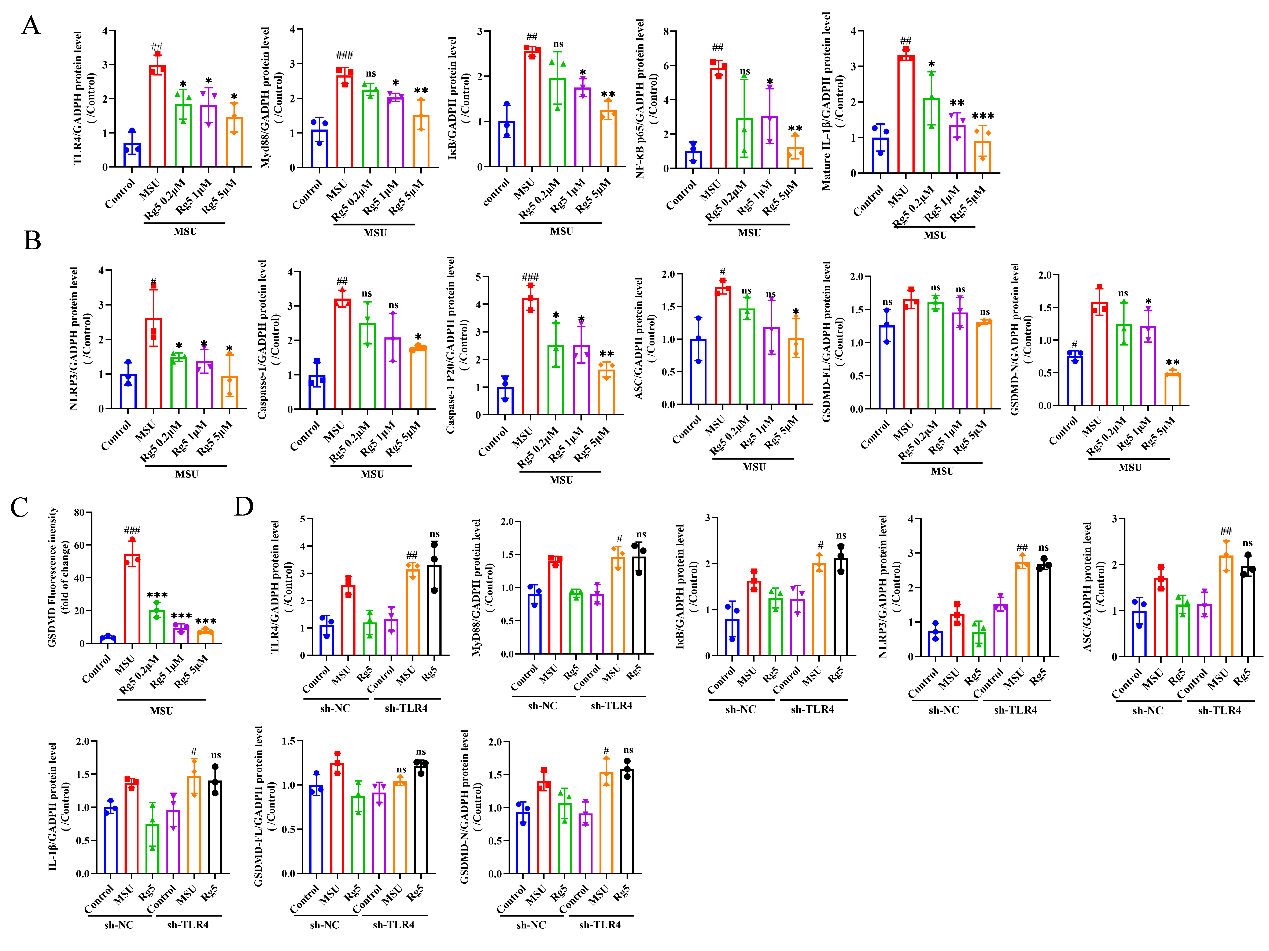


**Fig. S3. Validation of NOX1 treatment.** HK-2 was transfected with sh-NC (pcDNA3.1) or sh-NOX1 (pcDNA3.1(+)-human NOX1) for 48 hr and then stimulated with MSU for 24 hr (A-B). HK-2 was treated with or without ML171 (5 μM) for 3 h, followed by Rg5 and MSU for 24 h (C-D). (A-B) The expression level of overexpressed NOX1 was assessed by immunofluorescence (scale bar, 20μm), n=3. (C-D) Expression levels of NOX1 were assessed by immunofluorescence with and without the addition of ML171 (scale bar, 20 μm), n=3. ###P<0.001 vs. Control group; ***P<0.001 vs. MSU group.


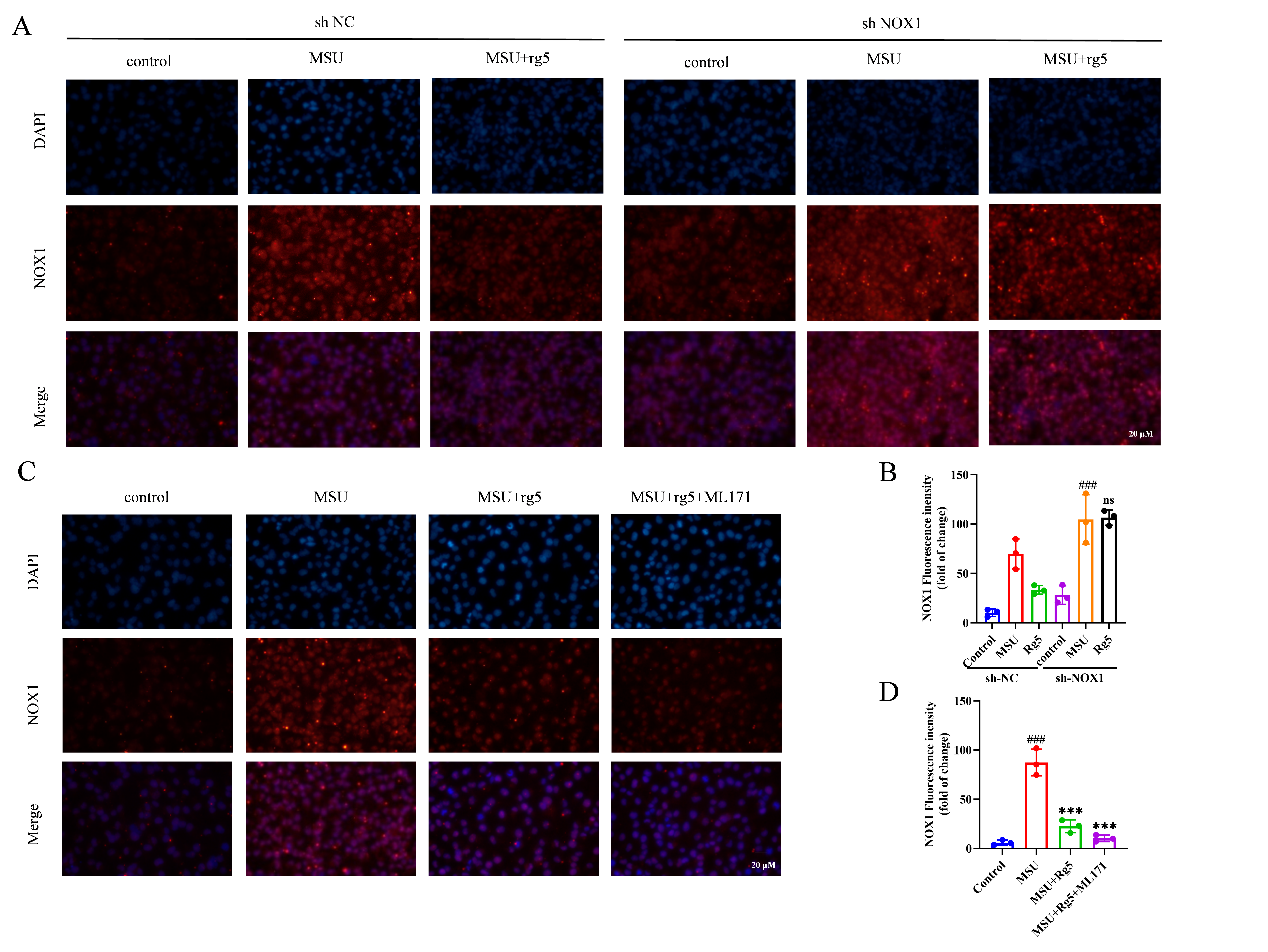


**Figure S4. Some statistics in Fig. 3.** (A)Changes in the relative protein expression levels of NOX1, TLR4, MyD88, IĸB, NF-ĸB P65, GSDMD-FL and GSDMD-N, n=3. (B) Changes in the relative protein expression levels of NOX1, n=3. (C, E) IF analysis of GSDMD expression, n=3. (D) Changes in the relative protein expression levels of NOX1, TLR4, MyD88, IĸB, NLRP3, Caspase-1, Caspase-1 P20, ASC, mature IL-1β, Pro IL-1β, GSDMD-FL and GSDMD-N, n=3. #P<0.05, ##P<0.01, ###P<0.001 vs. Control group; *P<0.05, **P<0.01, ***P<0.001 vs. MSU group.


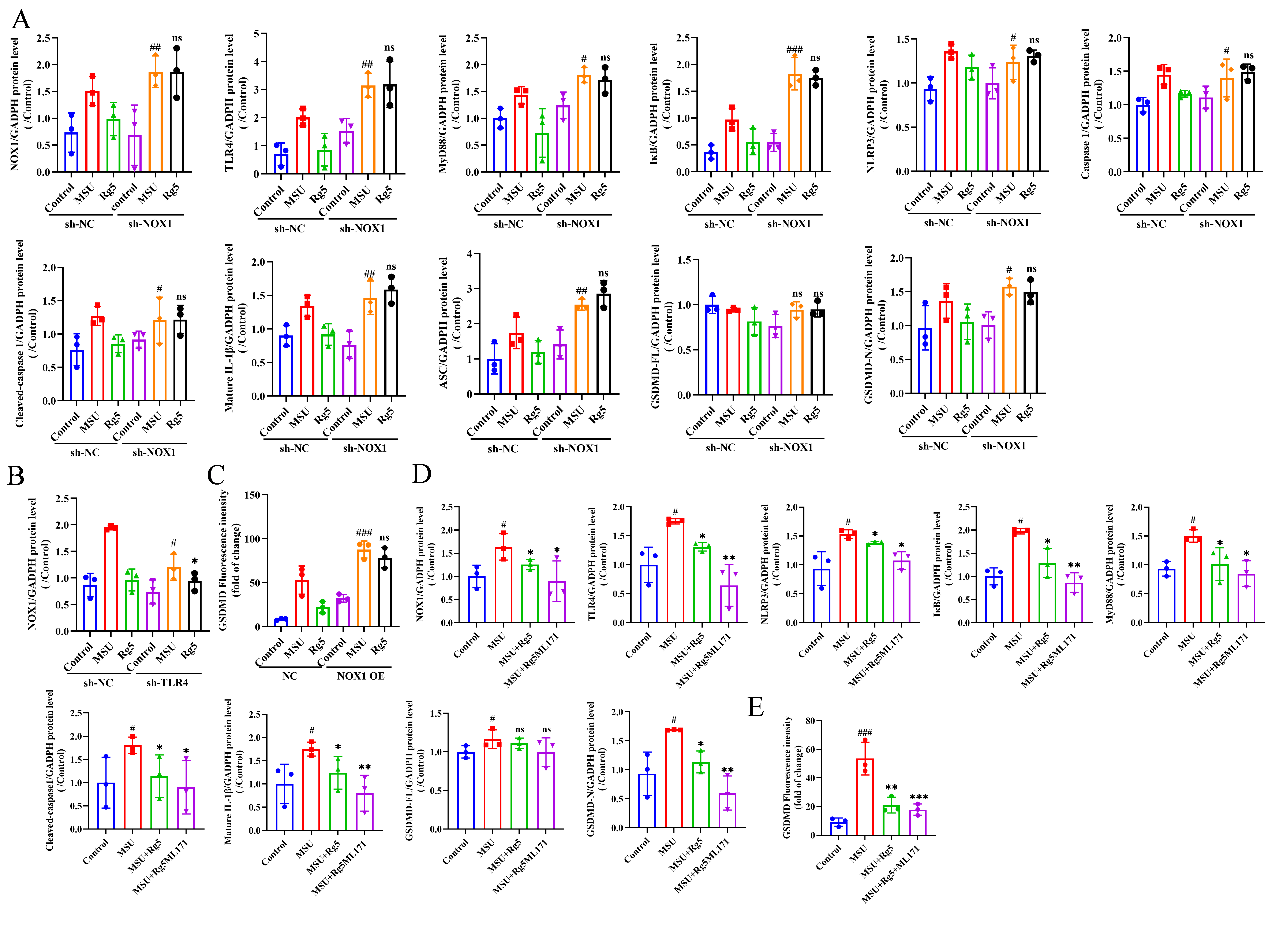


**Fig. S5. Resatorvid was unable to affect the inhibition of NOX1 by Rg5**. HK-2 was treated with or without Resatorvid (10 μmol/l) for 1 h, followed by Rg5 and MSU for 24 h. (A) Changes in the relative protein expression levels of NOX1, TLR4, MyD88 and IĸB, n=3. (B)Changes in the relative protein expression levels of NOX1, TLR4, MyD88, IĸB, n=3. #P<0.05, ##P<0.01, ###P<0.001 vs. Control group; *P<0.05, **P<0.01 vs. MSU group.

**
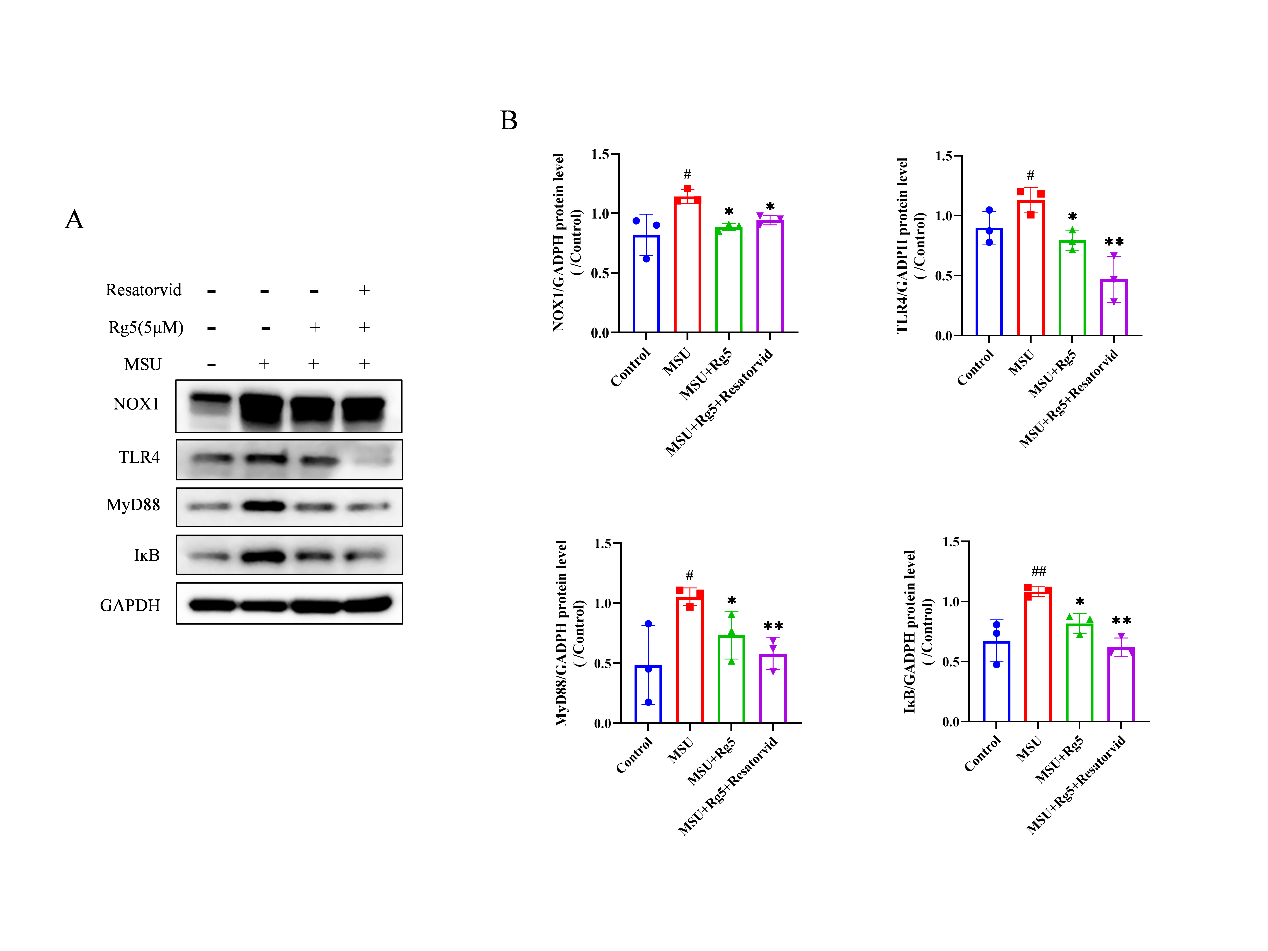
**

**Fig. S6. Changes in relative protein expression levels in Fig 5.** (A-B) Changes in the relative protein expression levels of P53, BCL-2 and BAX, n=3. #P<0.05, ##P<0.01, ###P<0.001 vs. Control group; *P<0.05, **P<0.01 vs. MSU group.


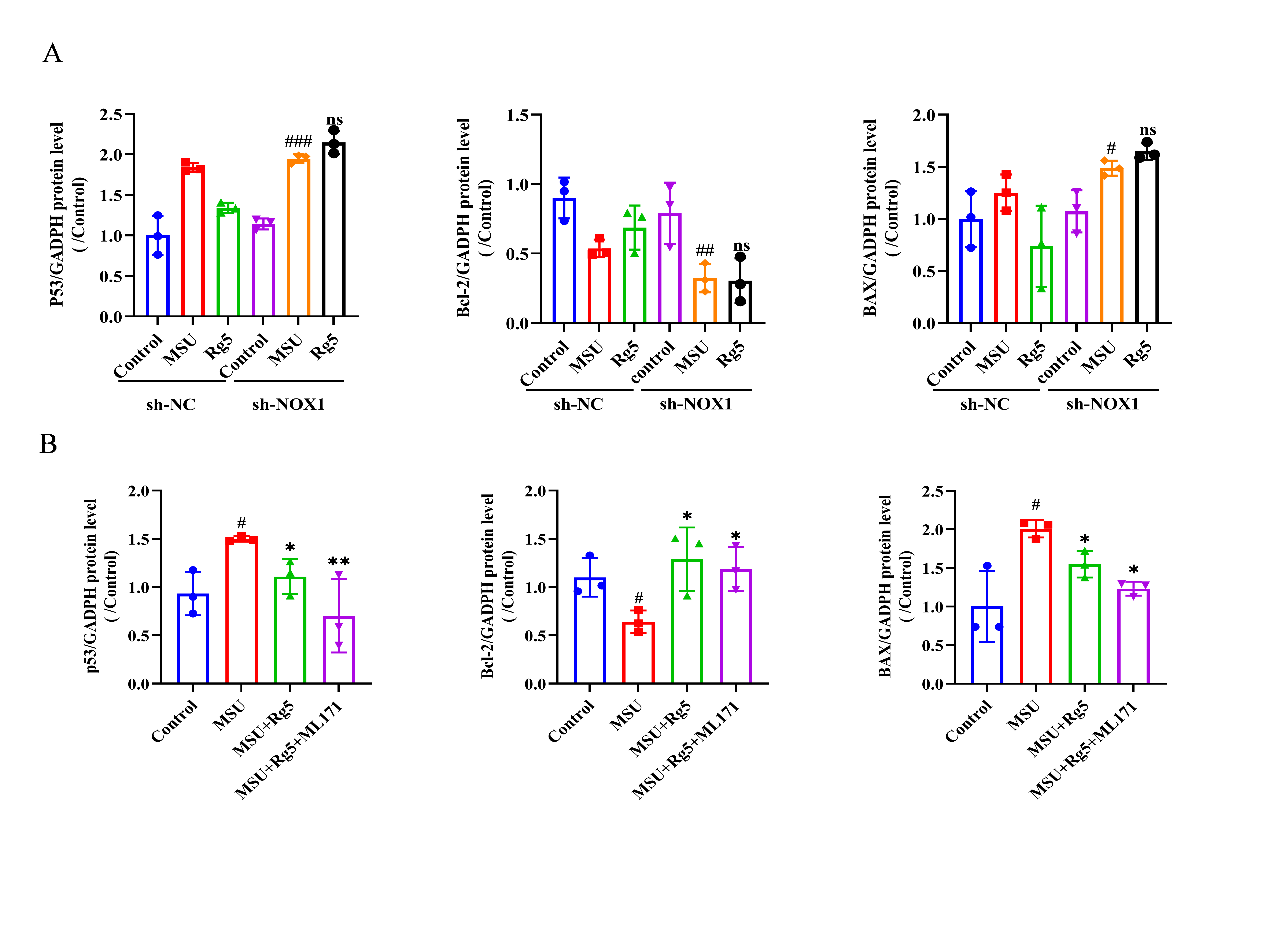


**Figure S7. Changes in the relative protein expression levels in Fig 6.** (A) Changes in the relative protein expression levels of ABGC2, OAT1, OCT2, URAT1, and GLUT9, n=3. (B-C) Changes in the relative protein expression levels of NXO1, TLR4, MyD88, IĸB, NLRP3, Caspase-1, Caspase-1 P20, ASC, mature IL-1β, Pro IL-1β, BCL-2, BAX, P53, GSDMD-FL and GSDMD-N, n=3. #P<0.05, ##P<0.01, ###P<0.001 vs. Control group; *P<0.05, **P<0.01, ***P<0.001 vs. MSU group.


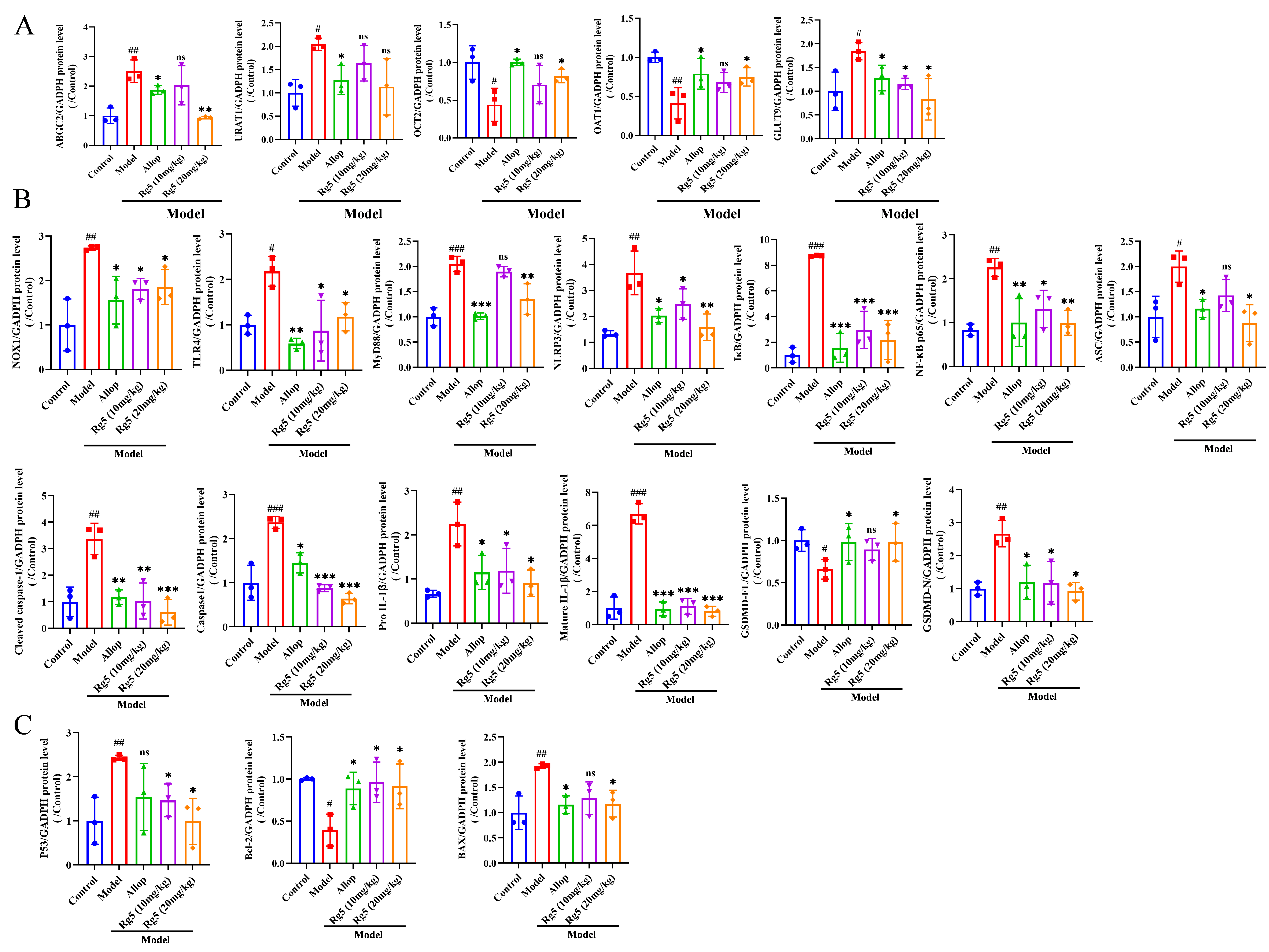

Supplement: Multimedia component 1 [file mmc1.docx]
